# Using mobile technology to support lower-salt food choices for people with cardiovascular disease: protocol for the SaltSwitch randomized controlled trial

**DOI:** 10.1186/1471-2458-14-950

**Published:** 2014-09-12

**Authors:** Helen Eyles, Rebecca McLean, Bruce Neal, Robert N Doughty, Yannan Jiang, Cliona Ni Mhurchu

**Affiliations:** National Institute for Health Innovation, University of Auckland, Private Bag 92019, Auckland, 1142 New Zealand; George Institute for Global Health, University of Sydney, PO Box M201, Missiden Road, Sydney, NSW 2050 Australia; Dept of Medicine, University of Auckland, Private Bag 92019, Auckland, 1142 New Zealand

**Keywords:** Cardiovascular diseases, Heart diseases, Salt, Sodium, Cellular phone, Smartphone, Telemedicine, Self-care, Secondary prevention

## Abstract

**Background:**

Cardiovascular disease (CVD) is the leading cause of early death worldwide, responsible for an estimated 29% of all global deaths. Reducing salt intake lowers blood pressure and risk of secondary cardiac events. However, identifying low salt foods can be challenging. SaltSwitch is a simple smartphone application (app) that enables shoppers to scan the barcode of packaged foods and receive an immediate, interpretive, traffic light nutrition label on the screen, along with suggestions for healthier lower-salt alternatives. A growing body of evidence suggests mobile technologies can support healthy behaviour change. However, robust evidence for the impact of smartphone interventions is lacking. This manuscript outlines the rationale and methods for a randomized controlled trial designed to determine the effectiveness of SaltSwitch in supporting people with CVD to make lower-salt food choices.

**Design/Methods:**

A 6-week, two-arm, parallel, randomized controlled trial is being undertaken in New Zealand (2 weeks baseline and 4 weeks intervention). Three hundred adults aged 40 years and older with CVD and their main household shoppers are recruited from research lists, cardiac rehabilitation clinics, and communities in Auckland. Participants are randomized to receive either the SaltSwitch smartphone app or no intervention (control). Randomisation is stratified by ethnicity and age. The primary outcome is the salt content of household food purchases. Secondary outcomes are the saturated fat and energy content of household food purchases, household food expenditure, use and acceptability of the SaltSwitch app by shoppers, and urinary sodium and blood pressure of participants with CVD. Ambulatory blood pressure and potential longer-term impact (12 weeks) of SaltSwitch will be assessed in sub-studies (n ~ 40 and n ~ 20, respectively). Household purchases of salt and other nutrients will be assessed using till receipt data electronically linked with branded food composition data.

**Discussion:**

The results of the SaltSwitch trial will determine the effectiveness, use and acceptability of a smartphone application to support lower salt food choices and secondary prevention of CVD.

**Trial registration:**

ACTRN12614000206628. Registered 30 March 2014.

## Background

Cardiovascular disease (CVD) is the leading cause of early death worldwide, being responsible for an estimated 15.6 million or 30% of all global deaths in 2010 [1]. In 2010, the number of years of life lost (YLLs) due to CVD and circulatory diseases was more than 273 million globally [1]; in New Zealand, CVD accounted for 17.5% (~167,000) of annual disability adjust life years, and Māori (indigenous New Zealanders) and Pacific peoples, and those living in lower socio-economic areas were disproportionately affected [2].

Secondary prevention is a vital strategy to reducing the global and national CVD burden, as 60% of hospitalisations occur in people who have been admitted for CVD in the past five years [3]. For people with CVD, adherence to a heart healthy diet has benefits additive to drug therapy and is associated with a reduction in mortality of between 8 and 45% [4–7]. Salt reduction is a key component of a heart healthy diet yet global and national salt intakes, particularly in high income countries, are far above recommended guidelines [8]. In New Zealand, population salt intake is ~9 g/day [9], almost twice the 2012 WHO recommendation and the recommended upper level of intake in NZ (5 g/day) [10, 11].

Cardiac rehabilitation programmes that offer dietary advice to people with CVD are provided by hospitals and non-government organisations in many countries, including New Zealand. However, they are resource intensive and uptake is generally low, largely due to access issues [12]. Furthermore, many people with CVD find it difficult to identify low salt food choices because nutrition labeling is confusing [13]. Smartphone technology may offer a more accessible, cost effective way of delivering nutrition interventions to cardiac patients. In New Zealand approximately two-thirds of the adult population owns a smartphone [14], and ownership is similar by ethnicity and income [15, 16]. Moreover, thousands of health and wellness applications (apps) are available direct to the public. Therefore, it is likely that smartphones will become synonymous with the future of patient self-care [17, 18].

**Figure 1:**
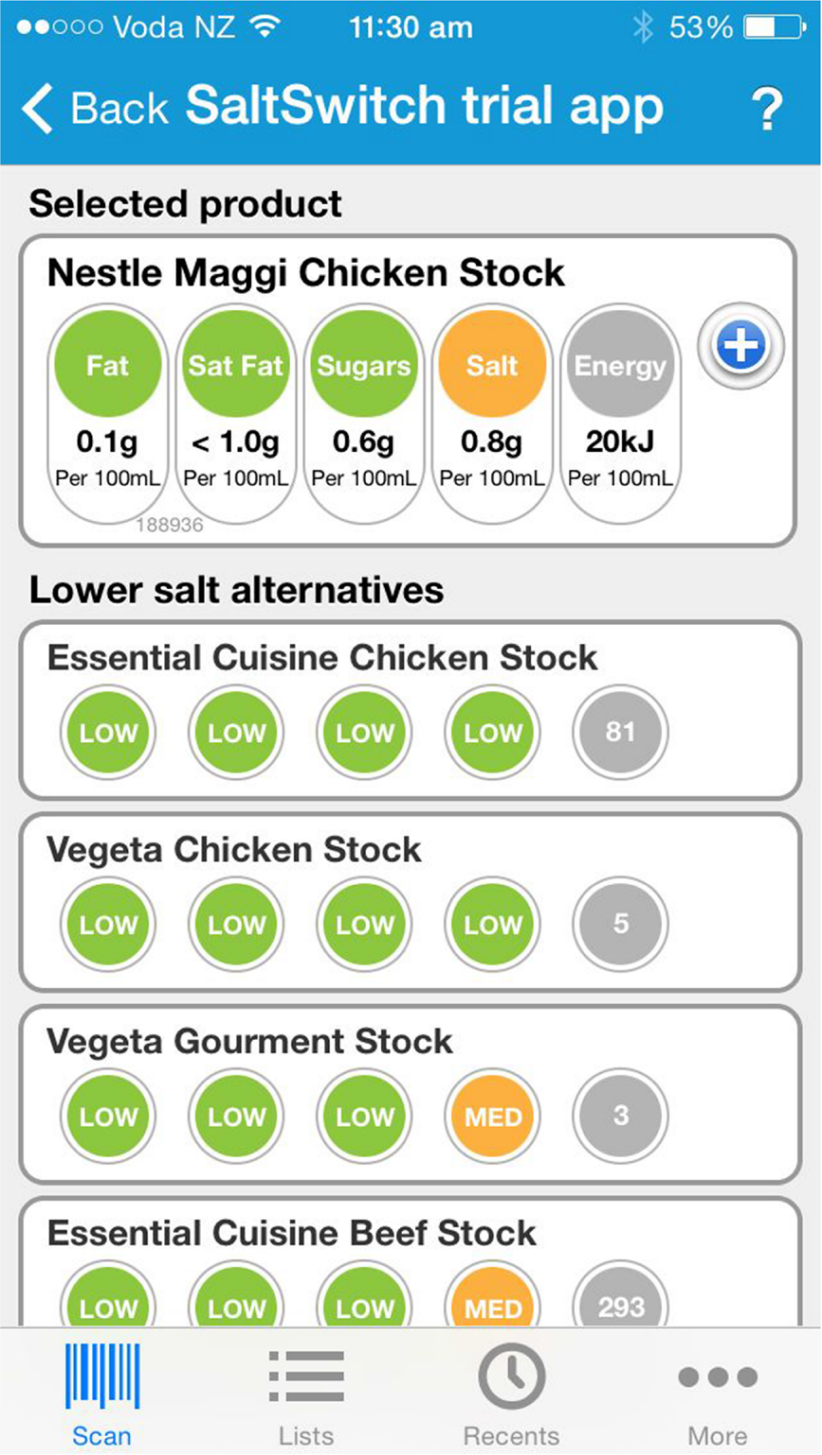
**The SaltSwitch smartphone application.**

SaltSwitch is a feature of the FoodSwitch smartphone app currently available in Australia, New Zealand, and the United Kingdom, designed to help consumers make healthier food choices [19]. FoodSwitch and SaltSwitch enable users to scan the barcode of packaged foods and receive an immediate interpretive, traffic light nutrition label on screen, along with a list of healthier lower-salt alternatives to ‘switch’ to (Figure 1). The main difference between FoodSwitch and SaltSwitch is an additional criterion in the algorithm used to determine healthier options [20]. The generic FoodSwitch algorithm allocates foods with a score based on their overall nutritional profile, and the additional criterion for SaltSwitch ensures that healthier alternatives are always lower in salt than the product scanned. SaltSwitch is not yet available in New Zealand, but it is available in the UK and Australia where it has proven functionality and consumer acceptability [21].

A growing body of evidence indicates that mobile technology can support behaviour change [22], yet robust evidence for smartphone interventions is lacking. This manuscript outlines the rationale and methods for a randomized controlled trial designed to determine the effectiveness of SaltSwitch in supporting people with CVD to make lower-salt food choices. The primary hypothesis is that four weeks’ intervention with SaltSwitch will reduce the salt content of household food purchases compared with control. Secondary hypotheses are that four weeks’ intervention with SaltSwitch will reduce the saturated fat and energy content of household food purchases, and the systolic blood pressure and salt intakes (measured by urinary sodium excretion) of participants with CVD. The SPIRIT checklist for Standardised Protocol Items was followed in writing this manuscript [23] and the protocol described reflects the most recent version (Version 2 dated 12^th^ May 2014).

## Methods/Design

### Trial design

**Figure 2:**
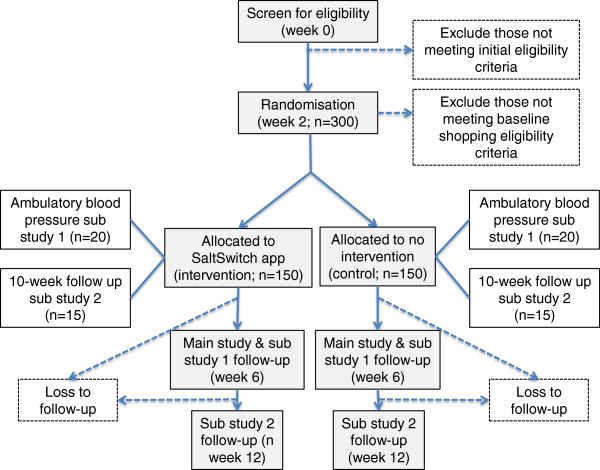
**SaltSwitch trial design.**

This study is a six-week, two-arm, parallel, randomized controlled trial (two weeks baseline and four weeks intervention; Figure 2) with a superiority design.

### Aims

The primary aim is to determine the effect of the SaltSwitch smartphone app on the salt content of food purchases made by households where at least one member has diagnosed CVD. Secondary aims are to evaluate the effect of SaltSwitch on: (1) saturated fat and (2) energy content of food purchases made by households; and (3) systolic blood pressure and (4) urinary sodium of participants with CVD. Household food expenditure, and use and acceptability of the SaltSwitch app by shoppers will also be assessed. Ambulatory blood pressure of participants with CVD and potential longer-term impact (12 weeks) of SaltSwitch will be assessed in two sub-studies (n ~ 40 and n ~ 20, respectively).

### Participants

Trial participants include individuals with CVD who complete most of the grocery shopping for their household, and pairs of individuals, one with CVD and the other who completes most of the grocery shopping. Only one person with CVD from each household may take part. Participants with CVD are men and women aged 40 years and older with a previous diagnosis of CVD (history of prior acute coronary syndrome, revascularization, or exertion angina). Main household shoppers (this may be the same person) must be 18 years and older, own or have access to a smartphone (iPhone or Android), and shop at the supermarket for household food at least once per week, spending $25 or more per household member.

Participants with CVD must not have suffered a cardiac event in the past three months, been diagnosed with heart failure or severe valve disease, be on a physician supervised diet or unwilling to make dietary changes, or be taking any medication that may lead to hyponatraemia or fluid retention (frusemide, regular non-steroidal anti-inflammatory (NSAID) use (excluding aspirin), or regular prednisone use). A cardiac event is defined as hospitalization for a heart attack, coronary artery revascularization, stroke, or heart failure. Main household shoppers must not currently use the FoodSwitch smartphone app or plan to use it during the next six weeks. All participants must not be planning on going away from home (vacation or otherwise) for more than three days in the next six weeks.

### Recruitment

Potentially eligible participants are invited to take part in the study via advertisements in supermarkets, hospital waiting rooms, healthcare organization newsletters, community newspapers, and other public spaces, and through social media (Facebook and Twitter). Research assistants also identify potential participants using an existing University of Auckland database comprising people with diagnosed CVD who previously agreed to be contacted for future research, a market research panel, and through cardiac rehabilitation clinics. Research assistants provide all potential participants with a summary of the trial over the phone to determine their interest in taking part. Those agreeing to participate are screened for eligibility and sent a study pack including a participant information sheet and consent form. Interested and eligible participants are allocated a unique study registration number including a household code (001 to 300), a number to indicate whether there are one or two people participating in the household (1 or 2), and a code to indicate whether they are the primary participant with CVD (1), or the secondary participant (main household shopper; 2). Participants with CVD are scheduled for a baseline assessment. Main household shopper participants who do not have CVD are sent a link in an email to complete an on-line questionnaire.

### Sample size

The target sample size of 300 participants with CVD (150 per group) will provide 80% power at 5% level of significance (two-sided) to detect a treatment difference of 0.07 g/MJ (~10%) of salt in household food purchases, between the two groups at follow up (average over weeks five and six) assuming a standard deviation of 0.22 g/MJ. The target sample size was estimated using data on baseline purchases of salt (mean (SD), 285 (88) mg/MJ) made by participants in a previous supermarket intervention trial (n = 1,104) in New Zealand [24] and estimated intervention effects from the Dietary Approaches to Stop Hypertension study [25].

### Ethical approval

Ethical approval was obtained from The University of Auckland Human Participants Ethics Committee on 16^th^ December 2013 (reference number 010998).

### Randomisation and blinding

Randomisation is undertaken at week two, following completion of the baseline period. During baseline, participants with CVD complete one clinic assessment (see measurement of outcomes section), and main household shoppers return all till receipts for food and non-alcoholic beverages to provide information on baseline shopping habits. Participants with CVD and their main household shoppers (this may be the same person) are excluded prior to randomisation if they spend < $25/household member per week on groceries/food or return less than two till receipts over the baseline period.

At the end of week two, eligible participants are randomized on a 1:1 ratio to receive either the SaltSwitch smartphone app or control (no intervention). Randomisation is stratified by ethnic (Māori and non-Māori) and age (40 to 59 years, and 60 years+) groups using variable block sizes. The randomisation lists are computer generated and sealed in individual opaque envelopes by an independent research assistant. The allocation sequence was generated and overseen by the study statistician (YJ). Envelopes are stored in a locked cabinet not accessible to the study statistician or Investigators. A study research assistant chooses a numbered envelope in sequential order from the correct stratum. Once allocated, the participant registration number is recorded on a sheet inside the envelope. Used envelopes are marked and placed at the back of the pile. Participants are telephoned to advise them of the group they have been allocated to.

Unique participant randomisation numbers ensure the study statistician is blinded to treatment allocation. However, due to the nature of the intervention it is not possible to blind participants or outcome assessors, and unblinding procedures are not relevant/required.

### Intervention

The intervention group receives the SaltSwitch smartphone app for four-weeks. SaltSwitch enables the user to scan the barcode of packaged foods and beverages using the phones camera and receive an immediate interpretive, traffic light nutrition label on screen, along with a list of healthier lower-salt alternatives to ‘switch’ to (Figure 1).

The New Zealand SaltSwitch app is underpinned by a database of ~13,000 barcodes and nutritional information for packaged foods and non-alcoholic beverages for sale in supermarkets and convenience stores [26]. Data are collected annually by fieldworkers [26]. SaltSwitch also incorporates crowd-sourcing; when a user scans the barcode of a product not already in the database, they are prompted to take three photographs (one each for the front of the pack, the nutrition information panel, and the ingredient list). Photographs are then sent to the research team and new products are subsequently added to the app.

The traffic light labels are determined using the United Kingdom’s Guidance for creating a front of pack nutrition label for pre-packaged foods [27], and healthier options are determined using the Australia New Zealand Food Standards Agency Nutrient Profiling Scoring Calculator (NPSC), a nutrient profiling model (scoring system to rank foods) used to determine whether foods are eligible to carry nutrition and health claims in Australia and New Zealand [20]. The NPSC system allocates a score to food products by balancing positive nutrients such as fibre and protein with negative nutrients including salt and saturated fat.

Main household shoppers in the intervention group are sent a link to download the SaltSwitch app to their smartphone, and are provided with a telephone tutorial. Intervention shoppers receive a reminder text message each week and are encouraged to use the app when shopping for household food and beverages.

### Control

Those randomised to the control group continue to shop as normal, and are able to access cardiac rehabilitation services as per usual care for people with CVD in New Zealand. Participants in the control group are advised on enrolment that they will be offered SaltSwitch on completion of the trial.

### Primary outcome

The primary outcome is the salt content of household food purchases (g/MJ) at the end of intervention (weeks five and six).

### Secondary outcomes

Saturated fat (g/MJ), energy content (kJ/MJ), and expenditure ($/MJ) on household food purchases will be assessed at the end of intervention (weeks five and six). Systolic blood pressure, ambulatory blood pressure (sub-study 1; n ~ 40), and urinary sodium of the household member with CVD, and use and acceptability of the SaltSwitch app (main household shopper) will be assessed at baseline (week 0) and end of intervention (week 6). With the exception of ambulatory blood pressure, all outcomes will be assessed over the longer-term, at weeks 11 and 12, in a sub-group of ~20 people with CVD (and their main household shopper where applicable) who remain enrolled in the study (sub-study 2). Serious adverse events (SAEs) will be recorded for the duration of the study.

### Measurement of study outcomes

Outcome measurements for participants with CVD are undertaken in clinics at one of two locations; either the University of Auckland, or Auckland Hospital. A research assistant telephones each participant to arrange a convenient time and venue of choice for baseline, week 6, and week 12 assessments (Figure 2). Main household shoppers who do not have CVD are also encouraged to attend. On arrival at the clinic, the researcher explains procedures, answers any questions, and collects consent forms (baseline only). Height, weight, blood pressure and a urine sample are collected, and participants self-complete two on-line questionnaires. Main household shoppers who are present at clinic appointments also self-complete baseline and follow-up questionnaires; those not present are sent a link to their questionnaire in an email.

Primary outcome: the salt content of household food purchases will be assessed using supermarket till receipts linked to brand-specific food and nutrient information from the SaltSwitch app (see Intervention section). Main household shoppers are provided with a receipt collection box and return address, postage paid envelopes to return all till receipts for household food and beverages purchased during the trial. All till receipts are photographed and uploaded to a bespoke web-based data entry system that stores data on all purchased foods and beverages and their costs. The number of purchased items not included in the SaltSwitch food and nutrient database will be recorded and included in analyses.

Secondary outcomes: the saturated fat and energy content of household food purchases, and expenditure on food, will be assessed using data from till receipts, as described for the primary outcome.

Weight, height, blood pressure and urine samples are collected using standard procedures. Weight is measured on a Salter scale to the nearest 0.1 kg and height is measured on a stadiometer to the nearest cm. Blood pressure of participants with CVD (mean of three readings taken at least 3 minutes apart) is assessed seated, after 10 minutes rest, using an OMRON automatic blood pressure monitor. Participants with an average blood pressure >160/90 mmHg, with an average systolic blood pressure <90 mm/Hg, or who report dizziness when going from sitting to standing, are advised to discuss this with their General Practitioner. Prior to commencement of the trial and annually thereafter, the Salter scales and OMRON automatic blood pressure monitor were serviced and verified according to manufacturer recommendations.

Participants in sub-study 1 assessing ambulatory blood pressure are provided a calibrated Mobil-O-Graph Ambulatory 24-hour Blood Pressure Monitoring System and instructions. Sub study 1 participants are instructed to wear the monitor for one 24-hour period within the following five days and keep a diary to record events such as bed and wake-up times. Ambulatory blood pressure will be analysed using programming software for the Mobil-O-Graph.

Twenty-four hour urinary sodium excretion is considered the gold standard for measurement of sodium intake [23]. However, 24-hour urine collections carry a high participant burden and are prone to under- and over-collection and low response rates [28]. In contrast, single urinary sodium collections carry a low participant burden and may provide a reasonable estimate of the sodium intake of a group [29]. Mid-stream urine samples are collected in standard clinical urine lab containers. Five millilitres of the urine sample is transferred to a labelled, 10 mL freestanding tube and stored in a secure −4°C freezer. Frozen samples are couriered in batches to a certified laboratory and converted to predicted 24-hour urinary sodium excretion using the INTERSALT formula [30].

Data on use and acceptability of the SaltSwitch app is collected from main household shoppers via an online questionnaire. The use of the app is assessed for each week of the intervention phase using a four-point scale ranging from ‘Used every time I shopped’ to ‘Not at all’. Acceptability is assessed via three questions. The first asks how easy the app was to use on a five-point scale from ‘Very easy’ to ‘Very difficult’; the second and third are open-ended questions asking what they liked most/least about the app, and whether or not they think the app is a good way to help people with CVD to make lower-salt food choices (and why). Data on serious adverse events are collected at six and 12 weeks, regardless of causal relationship to the treatment.

### Statistical analyses

Statistical analyses will be performed using SAS version 9.3 (SAS Institute Inc. Cary NC, USA). All statistical tests will be two-sided and a 5% significance level maintained throughout the analyses. Baseline characteristics will be summarised using descriptive statistics by treatment groups and overall. Continuous variables will be described as numbers of observed values, mean ± standard deviation. Categorical variables will be described as numbers and percentages. Since any differences between randomised groups at baseline could only have occurred by chance, no formal significance testing will be conducted.

Treatment evaluation will be performed on the principle of intention-to-treat (ITT), using data collected from all randomised participants. Missing outcome data will not be imputed, as it is difficult to make assumptions about the missingness of supermarket till receipt data, or the direction of the imputed values. Those randomised participants who are considered compliant to the study protocol will be included in a secondary per protocol (PP) analysis. Those households with at least three shopping episodes during the 4-week intervention period (seven or more shopping episodes during the 12-week intervention period for sub-study 2), with a total weekly spend of $25 or more per person, will be considered compliant. Analysis of covariance (ANCOVA) regression models will be used to evaluate the main treatment effect on the primary outcome, adjusting for baseline outcome measures; ethnicity, age, and gender. Model-adjusted means and their difference between the two treatment groups will be estimated and tested. A similar approach will be used for other continuous secondary outcomes, measured at the end of intervention period. Generalized linear models will be applied to categorical outcomes as appropriate. A pre-specified statistical analysis plan outlines all planned analyses and statistical methods.

## Discussion

The SaltSwitch trial is a robust randomized controlled trial of the short-term (four-week) effects of a promising mobile health intervention to reduce secondary heart disease. The trial is designed to maximize internal validity and is well-powered for the primary outcome (salt content of food purchases made by households). If found to be effective, SaltSwitch has potential to improve population health in New Zealand and globally; CVD is the leading cause of early death and disability worldwide [1], and population salt intakes in many countries far exceed WHO guidelines [8, 9, 31, 32]. Furthermore, the majority of salt consumed comes from packaged foods [33], which are the focus of the SaltSwitch app, and smartphone ownership is rapidly increasing, particularly in high income countries [14].

Nonetheless, the current SaltSwitch trial is not powered to explore effects amongst high risk groups in New Zealand, including Māori, Pacific, and those on low incomes. Moreover, the long term health impacts of the app for people with CVD and its effectiveness for primary prevention, are yet to be explored. However, these questions could be explored in future trials, and the findings of the current SaltSwitch trial will contribute significantly to the growing evidence base for the effectiveness of smartphone interventions; while there are several smartphone apps available on the market for people with CVD and other non-communicable diseases, the majority are not evidence-based [1, 34, 35].

## Authors’ information

HE is a Research Fellow and public health nutritionist; she is the principal investigator of the SaltSwitch trial. RM is a New Zealand Registered Dietitian and the principal SaltSwitch trial research assistant; BN is a Professor of Medicine and Chair of the Australian Division of World Action on Salt and Health; RND is the NZ Heart Foundation Chair of Heart Health; YJ is the senior biostatistician on the study; and CNM is a Professor of Population Nutrition; she is the Principal study co-investigator.

## Abbreviations

ANCOVA: Analysis of co-variance
CVD: Cardiovascular disease
DALY: Disability-adjusted life year
NSAID: Non-steroidal anti-inflammatory
PI: Principal Investigator
SAE: Serious adverse event
Smartphone app: Smartphone application
WHO: World Health Organization.
